# Recovery of Natural Hypoglycemic Compounds from Industrial Distillation Wastewater of Lamiaceae

**DOI:** 10.3390/molecules30061391

**Published:** 2025-03-20

**Authors:** Claudia Sciacca, Nunzio Cardullo, Martina Savitteri, Maria Gaetana Giovanna Pittalà, Luana Pulvirenti, Edoardo Marco Napoli, Vera Muccilli

**Affiliations:** 1 Department of Chemical Sciences, University of Catania, Viale A. Doria 6, 95125 Catania, Italy; claudia.sciacca@unict.it (C.S.); martina.savitteri@icloud.com (M.S.); mariagaetana.pittala@unict.it (M.G.G.P.); vera.muccilli@unict.it (V.M.); 2 Institute of Biomolecular Chemistry, National Research Council ICB-CNR, 95126 Catania, Italy; l.pulvirenti@icb.cnr.it

**Keywords:** phenolics, industrial waste, HPLC-UV, rosmarinic acid, macroporous resins, hypoglycemic agents, diabetes

## Abstract

The food industry generates the largest number of valuable by-products. The recovery of compounds such as fatty acids and polyphenols with notorious biological properties from biowaste is a new challenge in the circular economy scenario, as they represent value-added starting materials for the preparation of functional foods, food supplements, cosmetics and over-the-counter drugs. Less commonly explored are industrial wastewaters, which return to the nearby water streams without adequate treatment. Distillation wastewater (DWW) from the essential oils or agro-food industries may represent a valuable source of bioactive compounds to be valorized. In this work, DWW from rosemary was treated with different resins through dynamic and static adsorption/desorption approaches, for the recovery of phenolic compounds including rosmarinic acid. The most effective methodology, selected according to total phenolic and rosmarinic acid contents, as well as antioxidant activity evaluation, was applied to sage, thyme and oregano DWWs. The procedure provides several advantages compared with conventional separation processes, as it involves the lower consumption of reagents/solvents, low operational costs, ease of handling, and simplicity of scale-up. The results of this work highlight a fast and sustainable procedure for the recovery of rosmarinic acid and other phenolics (caffeic acid derivatives and flavonoid glycosides) from DWWS, thus affording a fraction with antioxidant and hypoglycemic activities.

## 1. Introduction

In 2020, the European Commission adopted the “new action plan for the circular economy”, one of the main components of the “European Green Deal” for sustainable growth [1]. This action, in line with the 2030 Agenda for Sustainable Development, adopted by all United Nations Member States, underlines the urgency, among other things, of promoting the circular economy and reducing waste production by supporting resource regeneration. When possible, biomasses are destined for energy recovery through the production of biofuels or methane. However, this is the least preferable fate for biomass, according to the waste hierarchy, which prioritizes waste reuse and recycling in various production cycles. In this context, the scientific community is increasing efforts to find alternative and sustainable ways to valorize food and agricultural waste, which could be rich sources of valuable or high-added-value compounds [2,3,4,5,6].

According to the Food and Agriculture Organization of the United Nations (FAO), the food industry generates among the largest amounts of by-products in the form of peels, kernels, pomace, unripe and/or damaged fruits and vegetables (FAO 2015). Plant and food biomasses are rich in polysaccharides (such as pectin and cellulose), enzymes (such as pineapple bromelain), and many other classes of secondary metabolites, such as polyphenols. Cellulose and other polysaccharides can be depolymerized into sugars, which can be converted into bioethanol, whereas the recovery of other compounds such as fatty acid and polyphenols is of importance for human health due to their biological properties (antioxidant, antimicrobial, anti-inflammatory, anticancer, etc.). Citrus peels were one of the first types of food industry waste utilized for the recovery of essential oils and flavonoids, and they are re-utilized as additives in food and fruit juices [7]. Olive kernels and grape pomaces are largely exploited as sources of polyphenols, and employed for the preparation of functional foods [8,9,10].

Less widely explored by-products include those connected to the wastewater of industries. It has been estimated that about 80% of the wastewater annually produced (380 trillion L) returns to nearby water streams without adequate treatment [11]. The representative example for the Mediterranean area is the olive mill wastewater, which is rich in organic compounds, such as phenols—well-known for their large array of biological activity, but highly polluting if released in industrial effluents, causing alterations in soil quality and phytotoxicity to aquatic life [12]. Only in the last decade has research been dedicated to the treatment of this type of biomasses to recover the (poly)phenolic fraction as a value-added product to be employed in food, cosmetic and nutraceutical fields, thus affording water suitable for irrigation purposes [13,14]. Other industrial activities have also focused on the extraction of vegetable matrices, and distillation wastewater (DWW) derived from the essential oils or agro-food industries may represent a valuable source of bioactive compounds to be valorized after they are recovered from wastewater. In a recent study from 2022, Truzzi et al. [15] demonstrated that the oil-exhausted biomass from *Lavandula angustifolia* and *L. intermedia* essential oil steam distillation represents an interesting source of bioactive compounds. The authors compared various extracts derived with different solvents, some of which (ethanol and water) are applicable on a large scale, showing their inhibitory effects on acetylcholinesterase and tyrosinase enzymes. Similarly, Fascella et al. analyzed the quality of essential oils and distillation waters of *L. angustifolia* samples on biochar, confirming that the distillation waters contain high amounts of bioactive compounds [16]. Navarrete et al. obtained fractions enriched in carnosol and carnosic acids through extraction from the residue of rosemary plants obtained after essential oil production [17]. DWW from basil, rosemary, and sage represents an exceptionally rich source of phenolic compounds, to be employed as an additive to prevent the oxidation and/or microbiological degradation of food, as well as functional ingredients for cosmetic, nutraceutical, and food applications [17,18]. Distillation biomasses from *Thymus vulgaris* are reported to be a source of bioactive compounds and polyphenols, especially rosmarinic acid (RA) and rutin, which also show hepatoprotective effects by reducing XOD xanthine oxidase activity in the production of superoxide radicals [17]. DWW from five *Lamiaceae* species cultivated in Sicily (*Rosmarinus officinalis* L., *Origanum vulgare* L., *Origanum majorana* L., *Salvia officinalis* L. and *Thymus vulgaris* L.) were investigated by Napoli et al. to assess their in vitro antioxidant and anti-inflammatory activities, with the aim of discovering useful information for their exploitation in the pharmaceutical and nutraceutical fields [19].

**Figure 1:**
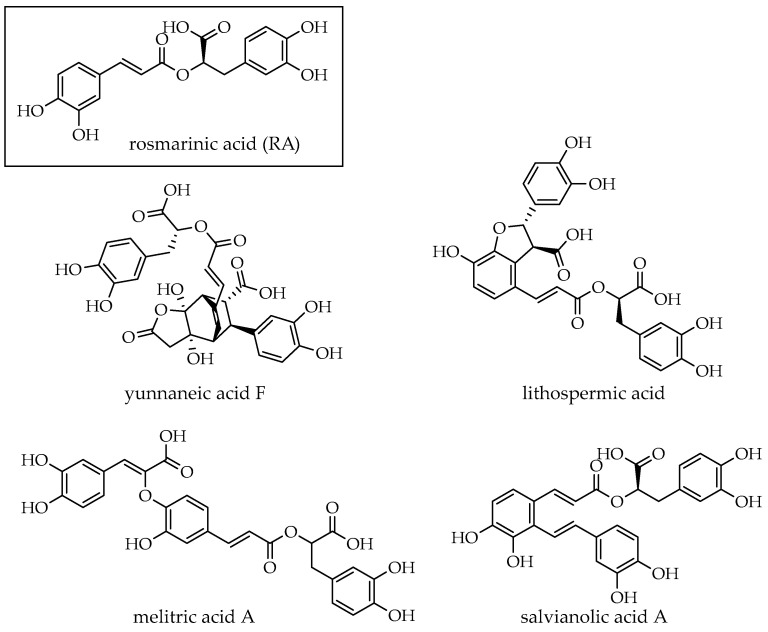
Chemical structures of RA and related compounds found in Lamiaceae.

Most of the properties listed above are attributed to rosmarinic acid, one of the main phenolic compounds isolated from *Lamiaceae* species such as thyme, rosemary, sage, and oregano. Rosmarinic acid, together with its derivatives (lithospermic acid, yunnaneic acid F, salvianolic acid A, and melitric acid A, Figure 1), has often been considered responsible for the health properties of these medicinal plants, including their anti-inflammatory, antioxidant, antitumor, antiviral, antimicrobial and hypoglycemic properties [20,21,22,23,24].

Often, the procedures for phenolic recovery from natural matrices based on solvent to solid extraction require long extraction times, high-energy dissipation, and the use of large volumes of organic solvents with low extraction selectivity. Although several eco-sustainable extraction processes have been developed over the years (ultrasound-assisted, microwave-assisted, supercritical fluid extraction), these are not suitable to be applied to wastewaters. The majority of works above-cited, highlighting the potential use of aromatic plants’ post-distillation waster, report data on the extraction from solid residues or on DWWs as they are, but none have tried to recover the phenolic fraction from this biomass. Adsorption resins are ideal for phenolic recovery from wastewaters; furthermore, they provide several advantages compared with conventional separation processes, as they entail the lower consumption of solvents if properly optimized, low operational costs and no energy consumption, they are easy to handle, and the processes are simple to scale-up [13]. Amberlite XAD resins are highly porous and cross-linked spherical polymers, very effective in the separation/purification of organic compounds present in plant and biological samples [25]. They are non-ionic resins and, based on the polymer matrix, they can be classified into two main groups: (a) polystyrene-divinylbenzene-based resins (XAD-1, XAD-2, XAD-4, XAD-16, XAD-1180, XAD-2000, and XAD-2010) and (b) polyacrylic acid ester-based resins (XAD-7, XAD-8, and XAD-11) [26]. They differ from one another by pore diameter, surface area, porosity, particle size, volume, etc. These characteristics directly influence the adsorption/desorption processes. Compared to other resins, such as ion-exchange resins, they can be easily regenerated without employing chemical reagents.

Among the above-cited resins, XAD-2, XAD-4, XAD-16 (non-polar) and XAD-7 (medium polar) are commonly used for the recovery of natural products, especially phenolic compounds, from complex mixtures or food industry waste, such as citrus and mango peel and apple pomace [27]. Depending on the structure of phenolics, with the presence or absence of glycosides, one resin may prove to be more effective than another; for instance, anthocyanins from mulberry were recovered more effectively with low polar XAD-7 than with XAD-5 [28]. Carnosic acid and carnosol were extracted with the highest yields when XAD-7 was employed [29], whereas XAD-16 showed very promising characteristics related to the recovery of catechin and ferulic acid [30].

In the present work, the best system for the recovery of phenolic compounds from DWW was defined. To this end, no extractive protocols have been used, but two different adsorption/desorption methodologies and four XAD resins (XAD-2, XAD-4, XAD-16, XAD-7), applied to rosemary distillation wastewaters, have been compared. The fractions were evaluated for polyphenol content, antioxidant activity, and extraction yield, and the data obtained allow the identification of the most efficient method. The best procedure was applied to DWW from sage, thyme and oregano. All the antioxidant fractions were evaluated for their potential hypoglycemic activity in light of the potential application of these new products as food ingredients and supplements for managing hyperglycemia. The innovation of this work lies in the use of solid phase extraction (SPE) directly on DWWS, a resource that has not been adequately exploited to date.

## 2. Results and Discussion

### 2.1. Recovery of Phenolics from Rosemary DWW and Antioxidant Activity

Distillation waste waters from rosemary (DWW-R) were used to optimize the procedure for the recovery of bioactive compounds, comparing two different SPE procedures and employing four different non-ionic XAD resins—XAD-2, XAD-4 and XAD-16 polystyrene-divinylbenzene based, and the polyacrylic XAD-7. These resins differ in porosity, surface area, and polarity. The two procedures applied are detailed in the materials and methods, and are herein summarized (see Table 1 for acronyms).

- Procedure A—Recovery by static adsorption/desorption: (i) mixing the DWW with resins, (ii) the discarding o the aqueous solution containing non-retained compounds (AP), and (iii) the desorption of retained compounds with ethanol (EP).
- Procedure B—Recovery by dynamic adsorption/desorption: (i) loading the DWW on the column packed with resins, and (ii) eluting with water (AF) and then with ethanol (EF).

The use of ethanol as a non-toxic solvent has been demonstrated widely; furthermore, it is an ideal solvent for maximizing polyphenols recovery.

The % yield of each procedure, the total phenolic content (TPC) and the antioxidant properties of DWW-R and related fractions are reported in Table 2.

A preliminary comparison was established between DWW-R and the aqueous samples R-AP and R-AF and the ethanol samples R-EP and R-EF. The ethanol samples obtained from all the resins showed comparable (EP) or higher TPC (EF) than DWW-R. Furthermore, the lowest TPC values recorded for the aqueous samples suggest that the phenolic constituents are reduced in water after the treatments. This trend was confirmed by antioxidant activity determination: ethanol samples showed higher antioxidant activities compared to the DWW-R sample, whereas the aqueous fractions were scarcely active. These results highlight two aspects, as follows: (i) the substantial content of polyphenols in DWW-R; (ii) solid-phase extraction applied directly on DWW, using water and ethanol as eluents, allows the almost-total recovery of phenolics from DWW.

In the literature, there are several procedures reported for the recovery of fractions of bioactive compounds from Mediterranean aromatic plant residues (solid and wastewater), most of them consisting of extraction from the solid residue with microwave-assisted extraction, ultrasound-assisted extraction, or using supercritical fluids. Only a few data are available on wastewater phenolic recovery by liquid–liquid extraction, pressurized liquid extraction or solid–liquid extraction, and even fewer by solid-phase extraction [31]. Ziani et al. obtained a TPC of 98.8 mg GAE/g from DWW-R recovered after steam distillation, but no enrichment procedure has been reported [32].

By comparing the results of the two procedures, it seems that the greatest antioxidant activity values and also the highest polyphenol contents (TPCs) were obtained with procedure B. Namely, the TPC increased in samples obtained with ethanol elution according to procedure B (71.3–146.8 mg GAE/g), when comparing DWW-R (63.9 mg GAE/g), while no significant variations are observed for the ethanol samples obtained with procedure A (42.2–72.2 mg GAE/g). This could be explained by the partial degradation of the phenolic constituents responsible for the antioxidant activity due to the long extraction time during procedure A (72 h) [33].

Correlation analysis was conducted to assess the direct correlation between TPC and the antioxidant activity measured according to DPPH, ABTS, and FRAP methods. As reported in Table 3, the radical scavenging activity measured by DPPH highly correlates with the TPC (*R*^2^ = 0.8597) and ABTS (*R*^2^ = 0.8481) results, but shows a lower correlation with FRAP (*R*^2^ = 0.5892 with *p*-value < 0.05). The antioxidant activity measured by the ABTS method highly correlates with the DPPH and FRAP (*R*^2^ = 0.8145) values, but shows a lower correlation with TPC values (*R*^2^ = 0.7507 with *p*-value < 0.01). In turn, the reducing metal power of the samples measured by the FRAP method shows a high correlation only with the data obtained from the ABTS assay, with the lowest correlation for TPC values (*R*^2^ = 0.54335 with *p*-value < 0.05). According to the TPC and antioxidant activity results, ethanolic fractions obtained from XAD-2 (R-EF2) and XAD-7 (R-EF7) have the highest content of phenolics. These findings agree with the data from the literature emphasizing the greater elution ability of polar compounds by XAD-2 with respect to XAD-16. Furthermore, the lower porosity and specific area of XAD-2 compared to XAD-16 enhances the interactions between the resin and the analytes. Moreover, XAD-7, with similar characteristics to XAD-2, but with medium polar character, allows the recovery of both polar and non-polar compounds such as flavonoids and cinnamic acids [25].

The analysis of the antioxidant results in relation to the resins employed is not fast enough, and therefore a multifactorial analysis was carried out, as detailed in the following.

### 2.2. HPLC-UV Quantification of Rosmarinic Acid in DWW-R and Fractions

The DWW-R and all samples were analyzed using HPLC-UV to quantify the content of rosmarinic acid (RA, Table 2), expressed in mg/g. The data show an RA content of 88.1 mg/g in DWW-R. Irakli et al. reported a lower RA content (between 51.40 and 58.93 mg/g) in a series of optimized extractions (ultrasound-assisted, microwave-assisted and accelerated solvent extraction [34]), emphasizing the greater effectiveness of the use of SPE directly on R-DWW compared to the application of extraction conditions to solid residues from the steam distillation of rosemary.

**Figure 2:**
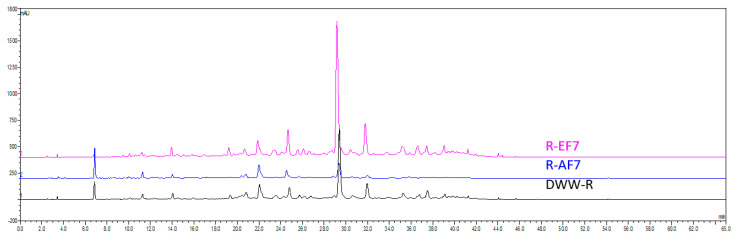
HPLC-UV profiles (330 nm) of DWW-R, R-AF7 and R-EF7.

Aqueous phases (AP) and fractions (AF) were characterized by a lower RA content compared to DWW-R, suggesting that the resins employed are able to adsorb rosmarinic acid, and ethanol allowed the desorption of the same. By analyzing the ethanol samples, it is possible to define the selectivity of the different resins in the adsorption/desorption of RA. For procedure A, a comparable recovery rate was observed, in the order of XAD-2 (94.3) ≤ XAD-4 (94.7) ≤ XAD-16 (95.0) < XAD-7 (97.7), indicating XAD-7 as the best resin. In procedure B, the recovery rate significantly increased in the following order: XAD-2 (90.8) < XAD-4 (95.3) < XAD-16 (96.6) < XAD-7 (99.8). The latter afforded a quantitative recovery of RA from DWW-R, thus defining it as the best procedure for this purpose. The chromatograms related to DWW-R and the fraction obtained with procedure B and XAD-7 are shown in Figure 2.

Procedure B presents obvious advantages in terms of implementation times, as the execution requires a couple of hours compared to the 72 h required in procedure A. Furthermore, the degree of ethanol consumption in procedure B is lower (6 mL/mL of DWW-R loaded) than in procedure A (10 mL/mL of DWW-R loaded). As such, the former procedure proved to be more sustainable than the latter, and allowed the highest degree of polyphenols recovery (according to TPC); XAD-7 in particular allowed the total recovery of RA.

### 2.3. Principal Component Analysis (PCA)

**Figure 3:**
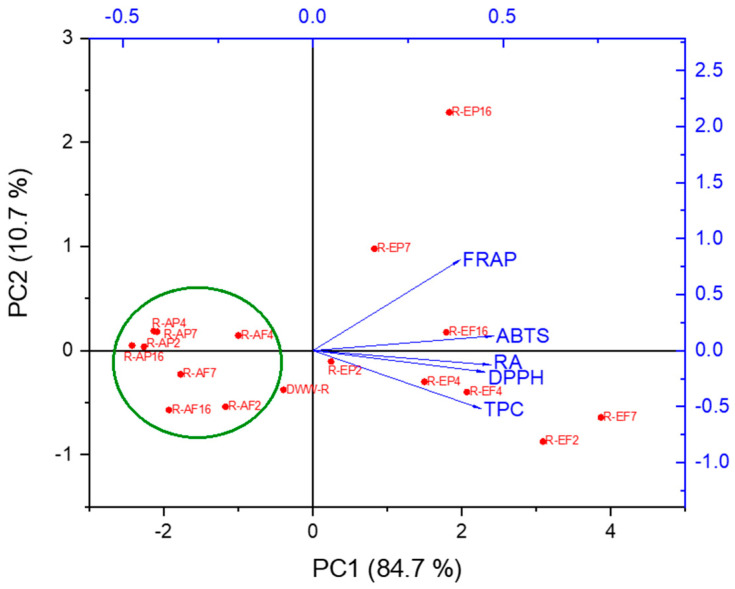
Biplot diagram according to principal component analysis considering the TPC, DPPH, ABTS, FRAP and rosmarinic acid content (RA) of DWW-R and its fractions (represented as points). The lines indicate the direction and magnitude of concentration for relevant variables.

Principal Component Analysis gave a general overview of data distribution, and so principal components (PCs) were generated. The PCA was performed with the data reported in Table 2 regarding the measurement of antioxidant activity, as well as the polyphenol and RA contents of the DWW-R, aqueous and ethanol samples obtained with all tested resins via the two procedures. The first principal component (PC1) showed the highest eigenvalue of 4.23594, and accounted for 84.7% of the total variance of the dataset. The second, third, and fourth PCs (PC2, PC3, and PC4) showed eigenvalue values of 0.53585, 0.17104 and 0.0394, respectively, representing 10.7%, 3.4% and 0.79% of the total variance, respectively. The larger the eigenvectors, the greater the correlations between variables and PCs (Table S1). The principal components PC1 and PC2 accounted for 95.4% of the total variance (Figure 3).

Following the analysis of the first two PCs, the following considerations were made. The TPC, DPPH, FRAP, ABTS and RA contents are all positively associated with PC1, and analogously all the ethanol samples (EP and EF) are located along the positive quadrant of PC1. PC2 positively correlates with FRAP and ABTS, and negatively with the results obtained from the other assays. The relationship between antioxidant assay measurements, TPC and ethanol samples can be easily seen from the PCA plot. PCA allows for the discrimination between the ethanol samples R-EP and R-EF (except for R-EP2) and the aqueous samples R-AP and R-AF, which clustered in the left part of the biplot (highlighted with green frame in Figure 3) and were negatively correlated with PC1. Among ethanol samples, R-EP16 could be significantly distinguished from other samples by its relatively high FRAP values, whereas R-EF2 and mostly R-EF7 exhibited relatively higher TPC, DPPH, ABTS and RA values than most of other samples, and were aligned to the right positive end of the PCA plot. Based on these findings, the XAD-7 resin employed with procedure B was chosen for the extraction of polyphenolic components from sage (DWW-S), thyme (DWW-T) and oregano (DWW-O) wastewater distillation.

### 2.4. Recovery of Polyphenols and RA from DWW-S, DWW-T, DWW-O and Antioxidant Activity

Samples of DWW-S, DWW-T and DWW-O underwent extraction and enrichment of their polyphenol content using XAD-7 resin according to Procedure B. Table 4 reports the antioxidant activity values (μmol TE/g) for each sample, and the results of DWW-R, R-AF7 and R-EF7 are also included for comparative analyses. In Table 4, the RA content and the total phenolic content obtained by HPLC-UV quantification at 330 nm are reported as well.

These results demonstrate that the procedure employed is effective for the recovery of polyphenols from all DWWs examined; the resulting EF fractions from sage, thyme, and oregano have shown comparable antioxidant activities, higher than those measured for DWW-R. The activity can be related to the phenolic content quantified at 330 nm, namely, with RA. This latter compound was recovered in the EF samples of all Lamiaceae DWW with high selectivity (S-EF7: 99.8%, T-EF7: 96.3%, O-EF7: 96.6%). Of note, this simple and scalable procedure allows the achievement of a value-added fraction of interest for nutraceutical, cosmetic and food applications. Further, the RA contents obtained for sage (S-EF7: 299.6 mg/g) and oregano (O-EF7: 259.0) are higher than those reported for the ultrasound-assisted extract obtained on post-distillation Greek sage (79.57 mg/g) and Greek oregano (66.38 mg/g) [35], suggesting that SPE may preserve the RA content.

### 2.5. Evaluation of Inhibitory Activity Against α-Glucosidase and α-Amylase

Considering the promising capacities of plant polyphenols, including rosmarinic acid, to manage hyperglycemia [36,37,38,39], the raw samples DWW-R DWW-S, DWW-T, and DWW-O and all the fractions obtained with the optimized procedure were in vitro assayed for their inhibitory activity toward α-glucosidase and α-amylase. These enzymes, normally involved in carbohydrate metabolism, have become targets in the search for hypoglycemic agents for their post-prandial glucose lowering effects. The data obtained have been elaborated as IC_50_ values and are reported in Table 5.

The activity towards the enzymes followed the same trend as the antioxidant activity—DWW-S, DWW-T and DWW-O have comparable inhibitory activities, proving more active than DWW-R. The ethanol samples were far more potent inhibitors than both aqueous samples and wastewaters, corroborating that the procedure developed allowed the recovery and concentration of phenolics from DWW with promising hypoglycemic activity. The inhibitory power of EF7 fractions toward α-amylase (18.3–12.1 µg/mL) was also comparable to that of acarbose (17.9 µg/mL), an antidiabetic drug, employed herein as the positive reference. Furthermore, the samples showed an acarbose-like behavior, being more potent inhibitors of α-amylase over α-glucosidase. It is probable that the inhibition magnitude can be related with the presence of rosmarinic acid among other polyphenols. In fact, RA and extracts enriched in RA have previously been reported for their inhibitory activity toward the carbohydrates hydrolyzing enzymes [36]. The ethanol fraction from DWW-S (S-EF7) showed lower IC_50_ values toward α-glucosidase (76.7 µg/mL) and α-amylase (12.1 µg/mL), and was thus the most active sample. Of note, this fraction was also the one with the highest RA (299.6 mg/g) and polyphenols content (768.3 mg/g), corroborating that the antioxidant principles are also responsible for enzyme inhibition.

### 2.6. HPLC-MS/MS Analysis of DWW and Enriched Fractions After Extraction

**Figure 4:**
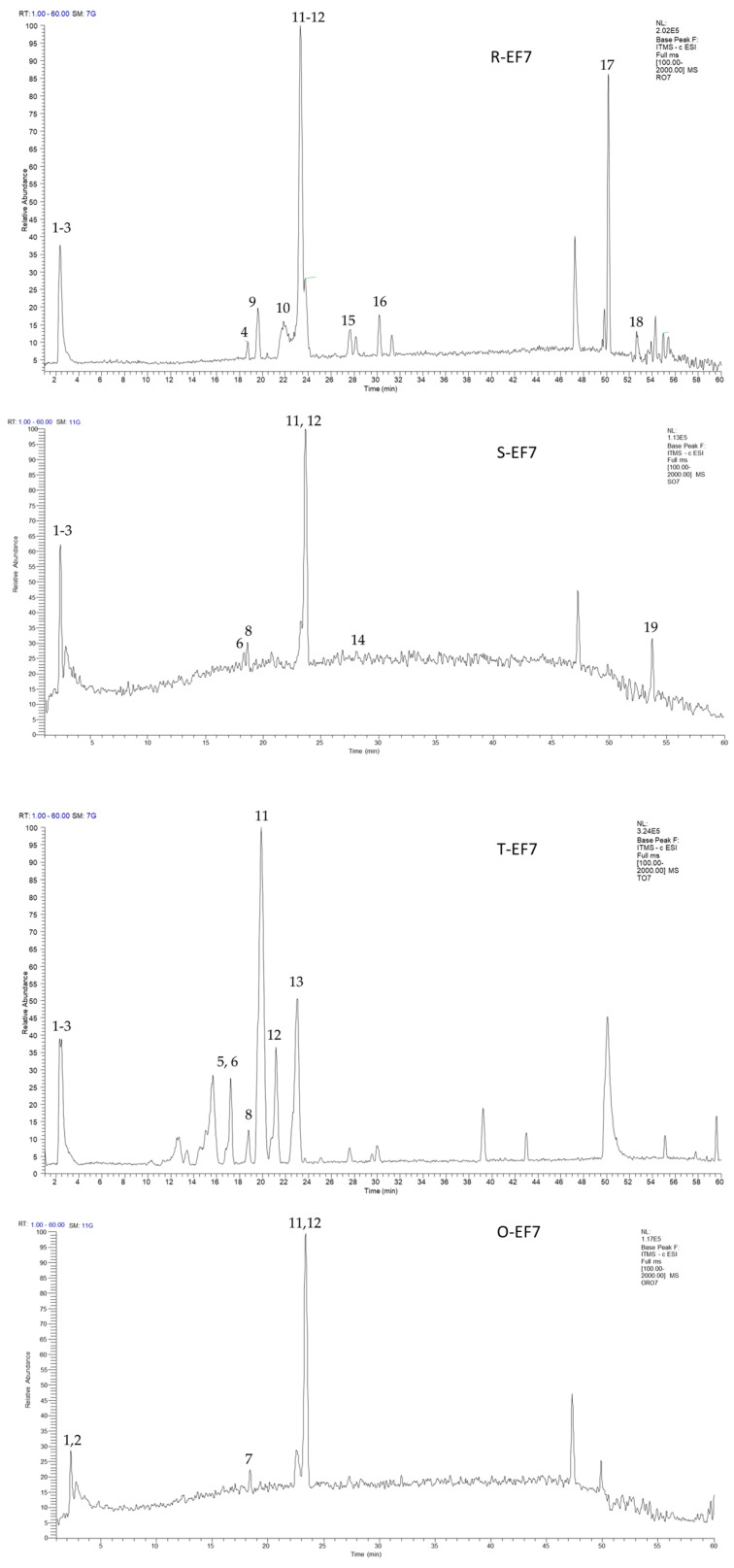
TIC chromatogram of ethanol fractions from DWWs of rosemary (R-EF7), sage (S-EF7), thyme (T-EF7) and oregano (O-EF7).

HPLC-MS analysis was employed to identify the components present in rosemary, sage, thyme, and oregano DWW samples, and their EF fractions. TIC chromatograms of EF are reported in Figure 4, while those of the corresponding DWWs are reported in Supplementary Materials (Figure S1).

The analyses were performed in negative ionization mode, and Table 6 reports the identified components for each sample along with their retention times. For each identified compound, the *m*/*z* value [M-H]^−^ and main MS/MS fragments are provided (see Table S2 for the identified compounds in each Lamiaceae). Component identifications were achieved by comparing the *m*/*z* values of the molecular ion and fragment ions of each component with the data in the literature. The chemical structures of identified compounds are reported in Figure S2.

The identified compounds can be grouped as follows: caffeic acid derivatives [caffeoyl glucose (**3**), yunnaneic acid F (**4**), salvianolic acid B/E isomer (**10**), rosmarinic acid (**11**), sagerinic acid (**12**), lithospermic acid (**13**), salvianolic acid A (**16**), rosmarichinone derivative (**19**)]; glucosylated flavonoids [quercetin-*O*-glucoside (**5**), luteolin-*O*-glucoside (**6**), luteolin 7-*O* glucuronide (**8**), isorhamnetin-*O*-glucoside (**9**), luteolin acetyl-glucuronide(**15**)]; organic acids [isocitric acid (**1**), malic acid (**2**)]; diterpenes [carnosol (**17**) and carnosic acid (**18**)].

All TIC chromatograms of DWW samples and their respective ethanol fractions show the most abundant component eluting at *t_R_* 23.5 min, corresponding to an [M-H]^−^ signal at *m*/*z* 359. This component has been identified as rosmarinic acid (**11**), and its fragmentation pattern exhibits signals at *m*/*z* 197, 179, 161, and 135 (Figure S3). The prevalent presence of this phenolic compound in DDWs and related ethanolic samples is in line with reports in the literature on post-distillation solid residue extracts from the same plants [35,40].

Caffeic acid derivatives were identified by a typical fragmentation pattern characterized by neutral losses of 198, 180, 162, 110, 44, and 18 Da, corresponding to the loss of 3,4-hydroxyphenyl lactic acid, caffeic acid, dehydrated caffeic acid, catechol, CO_2_, and water, respectively [21,41]. Other caffeic acid derivatives identified were yunnaneic acid F (**4**) with [M-H]^−^ at *m*/*z* 597, salvianolic acid B/E isomer (**10**) with [M-H]^−^ at *m*/*z* 717, lithospermic acid (**13**) with [M-H]^−^ at *m*/*z* 537, and salvianolic acid A (**16**) with [M-H]^−^ at *m*/*z* 493. The fragmentation pattern of **13** exhibits signals at *m*/*z* 493 (M-H-CO_2_), 359 (M-H-C_9_H_6_O_4_), 295 (M-H-C_9_H_10_O_5_-CO_2_) and 161 (M-H- C_9_H_6_O_4_-C_9_H_10_O_5_), confirmed by data in the literature [42]. The fragmentation patterns of these derivatives [43] are shown in Figure S4.

Of note, lithospermic acid (**13**) was found only in thyme samples (DWW-T and mostly in T-EF7); yunnaneic acid F (**4**) 5,6,7,10-tetrahydro-7-hydroxy rosmarichinone derivative (**19**) [44] was exclusive to the sage samples DWW-S and S-EF7, whereas salvianolic acid A (**16**) was present only in rosemary derivatives. Salvianolic acid B/E isomer (**10**) was identified in rosemary and thyme samples.

**Table 6:** Identification by HPLC-MS of the main constituents from 4 Lamiaceae distillation wastewaters and corresponding ethanol fractions.

	*t_R_* (min)	*m*/*z* [M-H]^− ^	Fragments *m*/*z* (Relative Intensity)	Identification	Reference
**1**	2.3	191	111 (100), 155 (10) 173 (30)	isocitric acid	[45]
**2**	2.3	133	115 (100), 87 (5)	malic acid	[46]
**3**	2.3	341	179 (100), 161 (20), 221 (20)	caffeoyl glucose	[47]
**4**	18.5	597	267 (20), 311.6 (100), 329 (40), 355 (20), 491 (20), 509 (20), 579 (20)	yunnaneic acid F	[47]
**5**	18.7	463	301 (100)	quercetin-*O*-glucoside	[48]
**6**	18.7	447	285 (100)	luteolin-*O*-glucoside	[48]
**7**	18.8	421	153 (100)	4-(3,4-dihydroxylbenzoyloxymethyl) phenyl-*O*-β-D-glucoside	[49]
**8**	19	461	285 (100)	luteolin-*O*-glucuronide	[50]
**9**	19.5	477	300 (10), 315 (100), 357 (5), 462 (5)	isoramnetin-3-*O*-glucoside	[50]
**10**	21.8	717	555 (5), 519 (100), 475 (20)	salvianolic acid B/E	[48]
**11**	23.5	359	161 (100), 179 (30), 197 (30), 135 (15)	rosmarinic acid	[50]
**12**	23.8	719	539 (45), 359 (100), 341 (35)	sagerinic acid	[47]
**13**	26.4	537	493 (100), 359 (10), 295 (5), 161(5)	lithospermic acid	[50]
**14**	26.8	555	493 (100), 359 (40)	salvianolic acid K	[48]
**15**	27.6	503	285 (100), 399 (20), 443 (5)	luteolin-3′-acetil-*O*-glucuronide	[48]
**16**	30.2	493	295 (100), 313 (10), 383 (5)	salvianolic acid A	[51]
**17**	50.1	329	285 (100)	carnosol	[52]
**18**	52.6	331	287 (100)	carnosic acid	[52]
**19**	53.8	345	301 (100)	5,6,7,10-tetrahydro-7-hydroxy rosmariquinone	[50]

Among the flavonoid glucosides, quercetin-*O*-glucoside (**5**), luteolin-*O*-glucoside (**6**), luteolin 7-*O*-glucuronide (**8**), isorhamnetin-3-*O*-glucoside (**9**), and luteolin-3-acetyl-*O*-glucuronide (**15**) were identified. A typical fragmentation pattern showed the presence of a hexose and the presence of aglycones at *m*/*z* 285, 301, and 315 corresponding to luteolin, quercetin, and isorhamnetin (Figure S5). Quercetin-*O*-glucoside (**5**), luteolin-*O*-glucoside (**6**) and isorhamnetin-3-*O*-glucoside (**9**) were present only in rosemary samples, whereas luteolin-3-acetyl-*O*-glucuronide (**15**) was identified only in R-EF7.

Isocitric acid (**1**) was unambiguously identified with an [M-H]^−^ signal at *m*/*z* 191, and its fragmentation patterns (sequential losses of *m*/*z* 173 (M-H-18), 155 (M-H-18-18), 111 (M-H-18-18-CO_2_)) allow for their discrimination from quinic acid, as reported in the literature [45]. Malic acid (**2**) was identified with an [M-H]^−^ ion at *m*/*z* 133.

The MS analysis of DWW-R and R-EF7 samples showed the presence of carnosol (**17**) and carnosic acid (**18**).

The identifications corroborate the presence of value-added compounds in DWWs, as most of the identified compounds have been well-studied for a large array of biological activities. Moreover, the presence in the ethanolic fractions and related DWWs of flavonoids and other caffeic acid derivatives, in addition to rosmarinic acid, can justify the notable hypoglycemic activity exerted by the samples, as these compounds have been reported to inhibit α-glucosidase and/or α-amylase.

## 3. Materials and Methods

### 3.1. Materials

Gallic acid, quercetin, stationary phases Amberlite XAD-16, XAD-7, XAD-4, and XAD-2, and 2,4,6-tripyridyl-s-triazine (TPTZ) were purchased from Merck (Milan, Italy).

Folin–Ciocâlteu reagent, 2,2-diphenyl-1-picrylhydrazyl radical (DPPH^•^), ferric chloride hexahydrate (FeCl_3_ 6H_2_O), and acetic acid (CH_3_COOH) were purchased from Fluka (Milan, Italy). Methanol (MeOH), ethanol (EtOH), hydrochloric acid (HCl), and sodium acetate trihydrate (CH_3_COONa 3H_2_O) were purchased from Carlo Erba (Milan, Italy).

6-Hydroxy-2,5,7,8-tetramethylchroman-2-carboxylic acid (Trolox) and ammonium salt of 2,2′-azino-bis(3-ethylbenzothiazoline-6-sulfonic acid) (ABTS^+^) were purchased from Sigma Aldrich (Milan, Italy). Pancreatic alpha-glucosidase for enzymatic assays and analytical-grade rosmarinic acid were purchased from Sigma Aldrich.

### 3.2. Plant Material

The medicinal plants under study (rosemary, sage, thyme, and oregano) were kindly provided by Azienda Agricola Rinoldo Davide, Via Gaspare Ambrosini 12, Favara (AG), Italy. The plants were collected between April and May 2021, each during its balsamic period (before complete flowering), and were dried in a ventilated and shaded place until a stable dry weight was obtained. For the hydrodistillation of oregano and thyme, their flowering tops were treated, while for rosemary and sage, the leaves were used, excluding the woody parts or twigs for each plant.

### 3.3. Hydrodistillation

The plant material (100 g) was treated with 1 L of distilled water in a round-bottom flask. It was connected to a Clevenger-type system for hydrodistillation, as previously reported [53]. After essential oil recovery, the distillation water (DWW), was filtered and frozen at −78 °C until use.

### 3.4. Solid-Phase Extraction (SPE)

Two different procedures were performed on the DWW, employing in both cases 4 XAD resins, as reported in the following (see Table 1 for acronyms). XAD-2, XAD-4, XAD-7, and XAD-16 resins were activated before use with EtOH (5 mL of EtOH per gram of resin) and kept in a shaker at 180 rpm for 1 h and at 37 °C.

Subsequently, the resins were filtered through filter paper, washed with deionized water (5 mL/g) and incubated at 180 rpm for 1 h at 37 °C.

- Procedure A. Recovery by static adsorption/desorption.

Here, 4 mL of DWW was added to 4 g of activated resin (XAD-2, XAD-4, XAD-7, or XAD-16) in a flask and mixed at 160 rpm for 24 h at 25 °C. The four mixtures were filtered through paper to recover the aqueous phase (AP) containing all the components of DWW not retained by the resin [54]. Subsequently, the filtered resins underwent the extraction of their adsorbed components by mixing with 20 mL of EtOH at 160 rpm, for 24 h at 25 °C [55]. This second filtrate represents the ethanol phase (EP) containing all the components of DWW retained by the resin and desorbed after the addition of EtOH. A further treatment with EtOH (20 mL) for 24 h was performed on each resin to eventually desorb any remaining component. Both AP and OP were dried under vacuum, kept in a desiccator until constant weight, and stored at −20 °C until use.

- Procedure B. Recovery by dynamic adsorption/desorption on column.

Here, 4 g samples of each resin (XAD-2, XAD-4, XAD-7, or XAD-16) suspended in EtOH were packed in a 1 cm column. Then, the resins were conditioned with water (with 4 times the bed volume). After that, 10 mL of DWW was poured into each column; after the adsorption, the column was eluted with water (40 mL) and the eluate was collected as an aqueous fraction (AF). Then, the column was eluted with EtOH (60 mL), thus affording the ethanol fraction (EF).

The fractions were dried, kept in a desiccator until constant weight, and stored at −20 °C until use.

### 3.5. Total Polyphenol Content (TPC) Evaluation

TPC was determined spectrophotometrically at 765 nm with a microplate reader (Biotek Synergy H1; Bad Friedrichshall, Germany), as previously reported [56]. Samples obtained from the different R-DWW treatments were solubilized in water:methanol (70:30 *v*:*v*) at concentrations ranging from 0.5 to 1.0 mg/mL. The results obtained are reported in Table 2 as mg GAE (gallic acid equivalents)/g of material.

### 3.6. Antioxidant Activity

The antioxidant activities of all samples were assayed with different methods (DPPH, ABTS, and FRAP).

DPPH. The DPPH radical scavenging activity was assayed as previously reported [57] on DWW, aqueous (AP and AF; 1 mg/mL in water) and ethanolic samples (OP and EF; 0.5 mg/mL in water:MeOH 70:30). Trolox solutions were prepared in methanol at different concentrations (10–80 μM). The UV-Vis data have been elaborated to give the % of radical quenched (Equation (1)). The linear regression analysis of the parentage of inhibition of Trolox vs. its concentration gave a calibration curve for expressing the inhibition of all the other samples in Trolox equivalents (µmol TE/g).

(1) $$quenched\;radical\;\left(\%\right)=\frac{OD_{radical}-OD_{sample}}{OD_{radical}}\;\times \;100\;$$

ABTS. The bleaching ABTS assay of DWW, AP, AF, OP and EF (0.1 mg/mL in water:MeOH 70:30) was performed as previously reported [57]. Trolox solutions were prepared in methanol at increasing concentrations (25, 50, 100, 150, and 200 μM) and assayed analogously. The linear regression of the percent of quenched radical vs. Trolox concentration allowed the elaboration of the samples’ results as TE.

FRAP. The ferric reducing antioxidant power was assessed for DWW, AP, AF, OP and EF (0.1 mg/mL in water:MeOH 70:30), as previously described [57]. Trolox samples (25–850 μM) were assayed under the same conditions, and the results of the samples are expressed as TE.

### 3.7. Enzyme Inhibition Assays

The α-glucosidase and α-amylase inhibitory potential of all DDW, AP, OP, AF and EF samples (1.0 to 0.5 mg/mL in water:MeOH 70:30) were assayed as previously described [58]. Acarbose was employed as a positive reference under the same conditions. The optical density acquired was elaborated to give the percentage of enzyme inhibition according to Equation (2). From the regression analysis of these data vs. sample concentration, the concentration that inhibited 50% of enzyme activity was calculated (IC50, µg/mL).

(2) $$enzyme\;inhibition\;\left(\%\right)=\frac{OD_{ES}-OD_{sample}}{OD_{ES}}\times 100\;$$

where OD_ES_ represents the optical density of the mixture containing the enzyme and the substrate, whereas OD_sample_ is the optical density of the same reaction but in the presence of the inhibitor (DDW, AP, OP, AF or EF).

### 3.8. HPLC-UV Analysis of Rosmarinic Acid

For HPLC-UV analysis, a Thermo Fisher (Madison, CA, USA) Ultimate 3000 HPLC-DAD was used, equipped with a Gemini 5 µm NX-C18 column (250 mm × 4.6 mm, 110 Å). The elution gradient of CH3CN acidified with 2.5% of formic acid (FA, solvent B) in H_2_O acidified with 2.5% of FA (solvent A) was optimized as follows, according to previous work [19]: t0 min B = 5.0%, t10 min B = 15%, t30 min B = 25%, t35 min B = 30%, t50 min B = 90%, t57 min B = 100% and t65 min B = 5%, with a flow rate of 1 mL/min. The analyses were recorded at three wavelengths—280 nm, 330 nm, and 350 nm. A pure sample of rosmarinic acid was used as an external standard to build a calibration curve (with concentration ranging from 0.062 to 0.744 mg/mL). The samples were injected (5 µL) at a concentration of 1 mg/mL.

### 3.9. HPLC-MS Analysis

Mass spectrometry analysis was performed using a Thermo Scientific (Madison, CA, USA) linear ion trap mass spectrometer LTQ equipped with an ESI ion source, coupled online with an HPLC system (Ultimate 3000, Dionex, Thermo Scientific, Madison, CA, USA). Each sample (10 mg/mL; 20 µL) was injected using an autosampler (Ultimate 3000, Dionex) into a Waters (Milford, MA, USA) Symmetry RP-C18 column (150 mm × 1 mm i.d., 100 Å, 3.5 μm). The chromatographic run was performed over 80 min at 50 µL/min—t0 min B = 5%, t25 min B = 15%, t40 min B = 25%, t55 min B = 55%, t60 min B = 95%, t65 min B = 100%, and t80 min B = 5%; solvent A consisted of H2O + 1% FA (formic acid) and B of CH3CN + 1% FA.

Two successive scanning modes were set, as follows: (1) Full-scan scanning in the range of 150–2000 *m*/*z*; (2) fragment ions scanning with a 2 Da isolation window. The following operational conditions were adopted: N_2_ gas flow rate 30 units/min; auxiliary gas flow rate (He) 8 units/min; spray voltage 4 kV; capillary voltage −18 V; capillary temperature 220 °C. Fragmentation was achieved with a collision energy of 29 a.u. using He as the collision gas. The mass axis was calibrated with a standard mixture consisting of caffeine, the peptide MRFA, and the polymer Ultramark. Data acquisition and processing were carried out using the Xcalibur software (v. 1.3) and the Qual browser interface (Thermo Scientific, Milan, Italy). The identification of constituents was manually inspected by analyzing their MS/MS spectrum and comparing these data with those from the literature.

## 4. Conclusions

This research has demonstrated that distilled wastewaters (DWWs) from four *Lamiaceae* species are rich in antioxidant and hypoglycemic compounds, particularly rosmarinic acid and other phenolic compounds. The study evaluated two methodologies and four resins for maximizing the recovery of value-added compounds from wastewater. The XAD-7 resin demonstrated the highest adsorption rate for rosmarinic acid, consistently yielding fractions with higher total phenolic content (TPC) and enhanced antioxidant and hypoglycemic activities compared to the original DWWs. The procedure developed here for recovering value-added compounds directly from industrial wastewater offers several advantages over conventional extraction methods. These benefits include reduced reagent and solvent consumption, lower operational costs, simpler handling procedures, and greater scale-up potential.

## Figures and Tables

**Table 1:** Acronyms adopted in the manuscript.

Sample	Resin	Procedure A	Procedure B	Acronym
Rosemary (R)				DWW-R
	XAD-2	aqueous phase		R-AP2
		ethanol phase		R-EP2
			aqueous fraction	R-AF2
			ethanol fraction	R-EF2
	XAD-4	aqueous phase		R-AP4
		ethanol phase		R-EP4
			aqueous fraction	R-AF4
			ethanol fraction	R-EF4
	XAD-7	aqueous phase		R-AP7
		ethanol phase		R-EP7
			aqueous fraction	R-AF7
			ethanol fraction	R-EF7
	XAD-16	aqueous phase		R-AP16
		ethanol phase		R-EP16
			aqueous fraction	R-AF16
			ethanol fraction	R-EF16
Sage (S)				DWW-S
	XAD-7		aqueous fraction	S-AF7
			ethanol fraction	S-EF7
Thyme (T)				DWW-T
	XAD-7		aqueous fraction	T-AF7
			ethanol fraction	T-EF7
Oregano (O)				DWW-O
	XAD-7		aqueous fraction	O-AF7
			ethanol fraction	O-EF7

**Table 2:** TPC, antioxidant activity (DPPH, ABTS and FRAP), % yield of process, recovery and rosmarinic acid (RA) content of DWW-R and all samples obtained from procedures A and B on different resins.

Samples	TPC (mg GAE/g)	Antioxidant Activity (μmol TE/g ± DS)			% Yield	RA (mg/g)
		DPPH	ABTS	FRAP		
DWW-R	63.9 ± 1.4 ^c^	15.4 ± 0.2 ^h^	60.9 ± 1.7 ^f^	29.3 ± 1.7 ^f,g^	-	88.1 ± 1.2 ^g^
Procedure A						
R-AP2	7.9 ± 1.2 ^g^	5.9 ± 0.1 ^j,k^	29.7 ± 2.2 ^h,i^	9.4 ± 1.2 ^i^	40.0	6.1 ± 0.1 ^i,j^
R-EP2	64.4 ± 9.5 ^c^	22.8 ± 0.1 ^g^	66.3 ± 7.3 ^f^	42.7 ± 1.5 ^e,f^	59.5	100.9 ± 2.4 ^f^
R-AP4	12.2 ± 0.8 ^g^	7.4 ± 0.1 ^j^	26.6 ± 2.3 ^h,i^	18.3 ± 2.6 ^g,h,i^	43.0	7.1 ± 0.2 ^i,j^
R-EP4	72.2 ± 8.8 ^c^	34.4 ± 1.9 ^e^	132.3 ± 0.1 ^e^	52.8 ± 15.3 ^d,e^	56.7	127.7 ± 0.1 ^d^
R-AP7	13.6 ± 1.9 ^f,g^	6.1 ± 0.1 ^j,k^	27.2 ± 0.2 ^h.i^	18.3 ± 2.6 ^g,h,i^	55.6	2.5 ± 0.1 ^j,k^
R-EP7	47.1 ± 2.3 ^d^	23.6 ± 1.5 ^g^	132.3 ± 2.1 ^e^	74.2 ± 5.6 ^b^	43.5	106.1 ± 0.4 ^e^
R-AP16	5.7 ± 0.2 ^g^	4.4 ± 0.1 ^k^	17.9 ± 3.7 ^i,j^	8.9 ± 0.2 ^i^	55.7	5.7 ± 0.1 ^i,j^
R-EP16	42.4 ± 6.2 ^d,e^	32.9 ± 0.2 ^e,f^	151.2 ± 8.3 ^d^	127.3 ± 1.3 ^a^	45.6	110.5 ± 0.6 ^e^
Procedure B						
R-AF2	46.1 ± 0.6 ^d^	23.9 ± 0.2 ^g^	35.2 ± 1.2 ^g,h^	14.7 ± 1.7 ^g,h,i^	46.3	18.0 ± 0.2 ^h^
R-EF2	126.8 ± 9.4 ^a^	51.4 ± 0.1 ^b^	187.7 ± 7.2 ^b^	54.5 ± 1.9 ^d,e^	33.4	178.0 ± 1.3 ^b^
R-AF4	15.6 ± 0.7 ^f,g^	31.2 ± 0.2 ^f^	65.8 ± 0.2 ^f^	26.6 ± 2.8 ^f,g,h^	74.5	8.3 ± 0.4 ^i^
R-EF4	94.9 ± 3.4 ^b^	42.7 ± 1.0 ^d^	130.8 ± 4.1 ^e^	57.2 ± 11.8 ^c,d,e^	26.6	169.2 ± 1.9 ^c^
R-AF7	28.2 ± 2.2 ^e,f^	12.3 ± 0.1 ^i^	45.4 ± 0.4 ^g^	10.5 ± 1.6 ^h,i^	55.8	0.4 ± 0.1 ^k^
R-EF7	134.5 ± 9.1 ^a^	55.0 ± 1.2 ^a^	204.1 ± 8.9 ^a^	71.2 ± 9.0 ^b,c^	39.0	219.0 ± 1.4 ^a^
R-AF16	31.6 ± 1.1 ^d,e^	17.2 ± 0.7 ^h^	9.1 ± 0.1 ^j^	3.9 ± 0.9 ^i^	42.7	3.4 ± 0.1 ^j,k^
R-EF16	71.3 ± 7.6 ^c^	47.2 ± 0.2 ^c^	164.9 ± 5.8 ^c^	62.4 ± 2.6 ^b,c,d^	34.5	99.0 ± 0.7 ^f^

Data are reported as means (*n* ≥ 3) ± SD. ^a–k^ Different letters in the same column indicate significant differences (Tukey’s test, *p* < 0.05).

**Table 3:** Pearson’s correlation coefficients of antioxidant activities and TPC.

	DPPH	ABTS	FRAP	TPC
DPPH	-	0.8481 ^a^	0.5892 ^b^	0.8597 ^a^
ABTS	0.8481 ^a^	-	0.8145 ^a^	0.7507 ^a^
FRAP	0.5892 ^b^	0.8145 ^a^	-	0.5435 ^b^
TPC	0.8597 ^a^	0.7507 ^a^	0.5351 ^b^	-

^a^ significant at *p* < 0.01; ^b^ significant at *p* < 0.05.

**Table 4:** Antioxidant activity (DPPH, ABTS and FRAP), % yield of the process, rosmarinic acid (RA) content and TPC (330 nm) of 4 Lamiaceae and fractions from XAD-7.

Sample	TPC (330 nm) mg/g	Antioxidant Activity (μmol TE/g ± DS)			Yield (%)	RA (mg(g)
		DPPH	ABTS	FRAP		
Rosemary						
DWW-R	152.5 ± 39.3 ^g^	15.4 ± 0.2 ^f,g^	60.9 ± 1.7 ^e,f^	29.3 ± 1.7 ^f^		88.1 ± 1.2 ^g^
R-AF7	3.3 ± 0.3 ^i^	12.3 ± 0.1 ^g^	45.4 ± 0.4 ^f,g^	10.5 ± 1.6 ^g^	55.8	0.4 ± 0.1 ^h^
R-EF7	399.2 ± 15.8 ^d^	35.5 ± 1.2 ^d^	204.1 ± 8.9 ^c,d^	71.1 ± 9.0 ^d^	39.0	219.0 ± 1.4 ^d^
Sage						
DWW-S	345.2 ± 8.1 ^e^	37.9 ± 0.7 ^c,d^	93.6 ± 1.9 ^e^	111.4 ± 10.5 ^c^		114.2 ± 0.4 ^c^
S-AF7	3.6 ± 0.1 ^i^	18.8 ± 0.2 ^f^	13.1 ± 3.6 ^g^	41.5 ± 1.7 ^e,f^	55.7	0.4 ± 0.1 ^h^
S-EF7	768.3 ± 12.5 ^b^	88.4 ± 1.6 ^a,b^	269.4 ± 12.4 ^b^	188.9 ± 14.8 ^a^	31.1	299.6 ± 0.3 ^a^
Thyme						
DWW-T	394.4 ± 4.7 ^d^	39.6 ± 0.8 ^c,d^	73.8 ± 2.8 ^e.f^	114.6 ± 6.5 ^c^		113.3 ± 0.3 ^e^
T-AF7	35.1 ± 0.3 ^h,i^	19.6 ± 0.6 ^f^	14.3 ± 0.2 ^g^	49.6 ± 2.5 ^e^	50.7	8.8 ± 0.2 ^h^
T-EF7	868.8 ± 19.7 ^a^	92.5 ± 1.0 ^a^	310.1 ± 7.3 ^a^	180.9 ± 6.1 ^b^	34.1	232.6 ± 5.0 ^c^
Oregano						
DWW-O	260.8 ± 3.6 ^f^	40.7 ± 0.2 ^c^	172.7 ± 8.9 ^d^	117.7 ± 4.8 ^c^		104.5 ± 0.2 ^f^
O-AF7	46.4 ± 0.3 ^h^	27.7 ± 1.1 ^e^	54.8 ± 7.2 ^f^	86.7 ± 9.6 ^d^	24.1	8.9 ± 0.6 ^h^
O-EF7	556.9 ± 4.7 ^c^	86.8 ± 3.6 ^b^	226.4 ± 27.1 ^c^	166.8 ± 4.4 ^b^	64.2	259.0 ± 0.7 ^b^

Data are reported as means (*n* ≥ 3) ± SD. ^a–i^ Different letters in the same column indicate significant differences (Tukey’s test, *p* < 0.05).

**Table 5:** Hypoglycemic activity, determined as in vitro α-glucosidase and α-amylase inhibition, of the DWW of 4 Lamiaceae and fractions obtained with procedure B and XAD-7.

Sample	α-Glucosidase	α-Amylase
DWW-R	643.2 ± 15.0 ^a^	73.9 ± 3.6 ^a^
R-AF7	460.4 ± 29.2 ^b^	66.5 ± 1.3 ^b^
R-EF7	96.7 ± 2.4 ^g^	18.2 ± 0.3 ^f^
DWW-S	272.3 ± 35.9 ^d^	35.7 ± 2.1 ^e^
S-AF7	663.7 ± 23.2 ^a^	43.8 ± 2.6 ^d^
S-EF7	76.7 ± 4.3 ^g^	12.1 ± 1.4 ^f^
DWW-T	196.1 ± 13.4 ^e,f^	39.7 ± 2.5 ^d,e^
T-AF7	216.7 ± 17.6 ^d,e,f^	34.9 ± 0.8 ^e^
T-EF7	83.4 ± 5.6 ^g^	18.3 ± 2.1 ^f^
DWW-O	250.3 ± 30.2 ^d,e^	50.8 ± 2.2 ^c^
O-AF7	391.7 ± 13.4 ^c^	43.5 ± 3.7 ^d^
O-EF7	80.6 ± 2.2 ^g^	17.3 ± 0.9 ^f^
Acarbose	161.4 ± 8.2 ^f^	17.9 ± 0.6 ^f^

Data are reported in IC_50_ (µg/mL) as means (*n* ≥ 3) ± SD. ^a–g^ Different letters in the same column indicate significant differences (Tukey’s test, *p* < 0.05).

## Data Availability

The original contributions presented in this study are included in the article/Supplementary Materials. Further inquiries can be directed to the corresponding authors.

## Supplementary Materials

The following supporting information can be downloaded at: https://www.mdpi.com/article/10.3390/molecules30061391/s1, Table S1: Extracted eigenvectors; Figure S1: TIC chromatograms of DWWs of rosemary (DWW-R), sage (DWW-S), thyme (DWW-T) and oregano (DWW-O); Table S2: Phytochemicals identified in the four Lamiaceae analyzed; Figure S2: Chemical structures of identified compounds; Figures S3–S5: Fragmentation pathways of identified compounds.
