# Supplementary material for: Recovery of Natural Hypoglycemic Compounds from Industrial Distillation Wastewater of Lamiaceae
Source: Molecules. 2025 Mar 20;30(6):1391. doi: 10.3390/molecules30061391 (PMC11944828; doi:10.3390/molecules30061391)
Supplement: Supplementary file 1 [file molecules-30-01391-s001.zip › molecules-3517660-supplementary.pdf]

Supplementary Materials

Recovery of Natural HypoglycemicCompounds from Industrial Distillation Waste Water of Lamiaceae

Claudia Sciacca,[1] Nunzio Cardullo,[1,*] Martina Savitteri,[1] Maria Gaetana Giovanna Pittalà,[1] Luana Pulvirenti,[2] Edoardo Marco Napoli,[2,*] Vera Muccilli[1]

[1] Department of Chemical Sciences, University of Catania, Viale A. Doria 6, 95125, Catania, Italy

[2] Institute of Biomolecular Chemistry, National Research Council ICB-CNR, 95126 Catania, Italy

Table S1. Extracted Eigenvectors

	**Coefficients of PC1**	**Coefficients of PC2**
TPC	0.44325	-0.5198
DPPH	0.45409	-0.19437
ABTS	0.47578	0.13174
FRAP	0.38838	0.81162
RA	0.46918	-0.12626

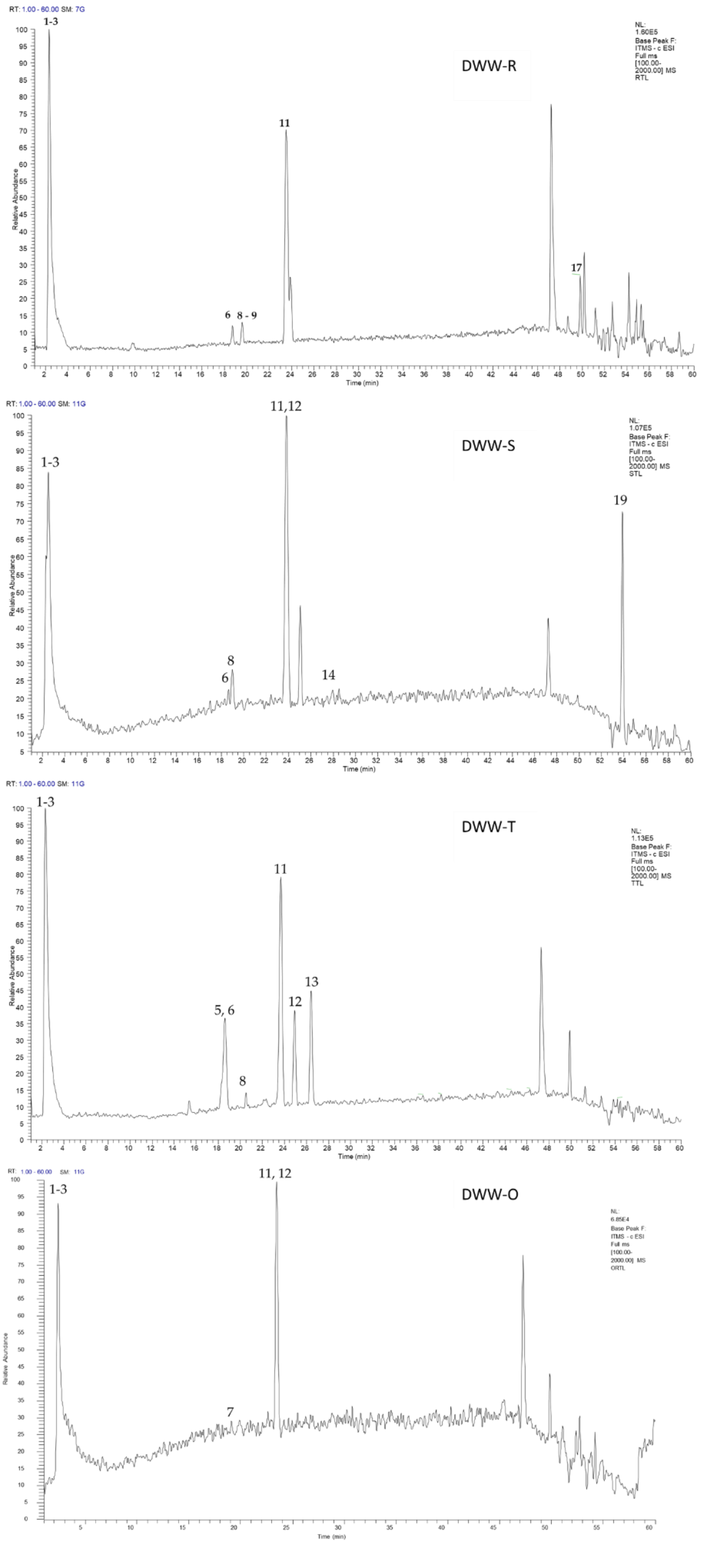

Figure S1. TIC chromatograms of DWWs of rosemary (DWW-R), sage (DWW-S), tyme (DWW-T) and oregano (DWW-O).

Table S2. Phytochemicals identified in the four Lamiaceae analysed

	Identification	rosemary			thyme			sage			oregano		
		DWW	AF	EF	DWW	AF	EF	DWW	AF	EF	DWW	AF	EF
**1**	isocitric acid	X	X	X	X	X	X	X	X	X	X	X	X
**2**	malic acid	-	-	-	X	-	X	X	-	X	X	-	X
**3**	caffeoyl glucose	X	X	X	X	X	X	X	X	X	X	X	-
**4**	yunnaneic acid F	X	X	X	-	-	-	-	-	-	-	-	-
**5**	quercetin-*O*-glucoside	-	-	-	X	-	X	-	-	-	-	-	-
**6**	luteolin-*O*-glucoside	-	-	-	X	-	X	X	-	X	-	-	-
**7**	4-(3,4-dihydroxylbenzoyl oxymethyl) phenyl -O-β-D-glucoside	-	-	-	-	-	-	-	-	-	X	X	X
**8**	luteolin-*O*-glucuronide	X	-	-	X	-	X	X	-	X	-	-	-
**9**	isorhamnetin-3-*O*-glucoside	X	-	X	-	-	-	-	-	-	-	-	-
**10**	salvianolic acid B/E	-	-	X	X	-	X	-	-	-	-	-	-
**11**	rosmarinic acid	X	-	X	X	-	X	X	-	X	X	-	X
**12**	sagerinic acid	-	-	X	X	-	X	X	-	X	X	-	X
**13**	lithospermic acid	-	-	-	X	-	X	-	-	-	-	-	-
**14**	salvianolic acid K	-	-	-	-	-	-	X	-	X	-	-	-
**15**	luteolin-3′-acetil-*O*-glucuronide	-	-	X	-	-	-	-	-	-	-	-	-
**16**	salvianolic acid A	-	-	X	-	-	-	-	-	-	-	-	-
**17**	carnosol	-	-	X	-	-	-	-	-	-	-	-	-
**18**	carnosic acid	-	-	X	-	-	-	-	-	-	-	-	-
**19**	5,6,7,10-tetrahydro-7-hydroxy rosmariquinone derivative	-	-	-	-	-	-	X	-	X	-	-	-

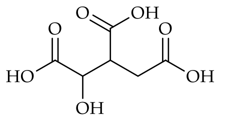

1

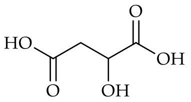

2

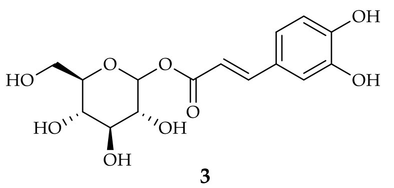

3

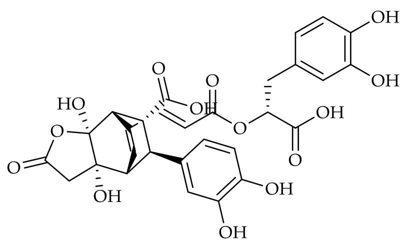

4

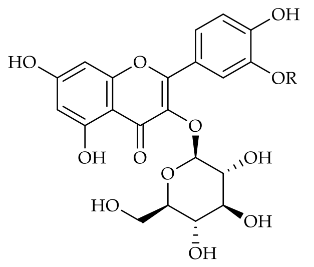

5 R = H
9 R = CH_3

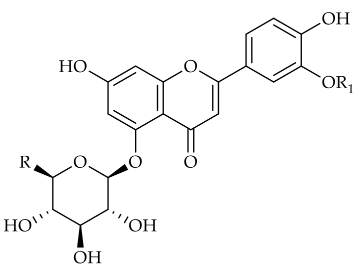

6 R = CH_2OH, R_1 = H
8 R = COOH, R_1 = H
15 R = COOH, R_1 = Ac

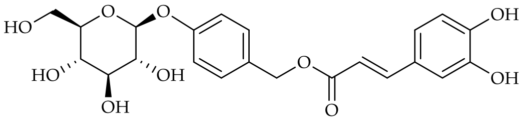

7

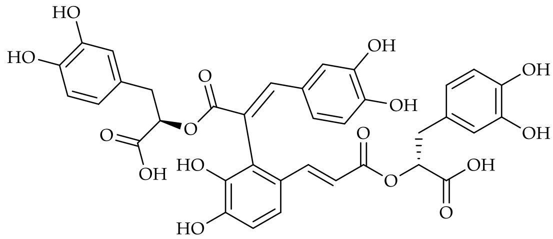

10 (salvianolic acid E)

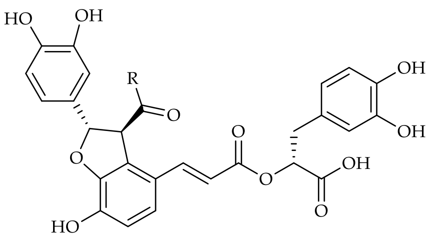

10 (salvianolic acid B) R = 3,4-dihydroxy phenyl lactic acid
13 R = OH

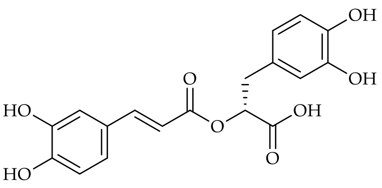

11

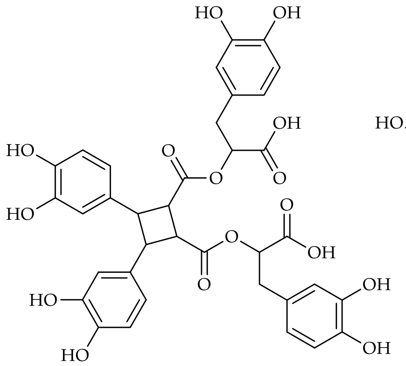

12

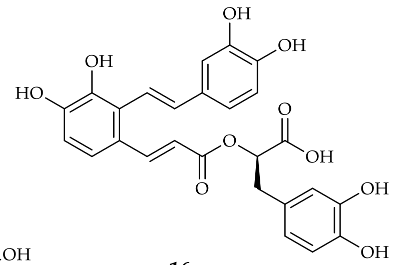

16

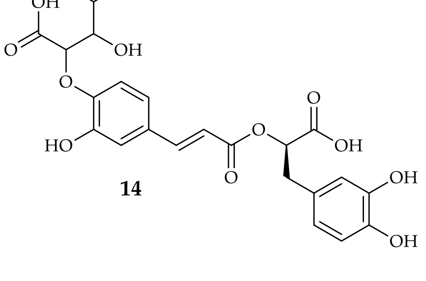

14

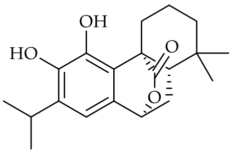

17

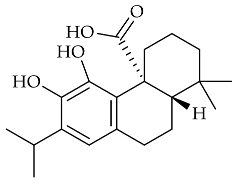

18

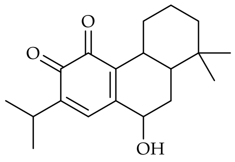

19

Figure S2. Chemical structures of identified compounds.

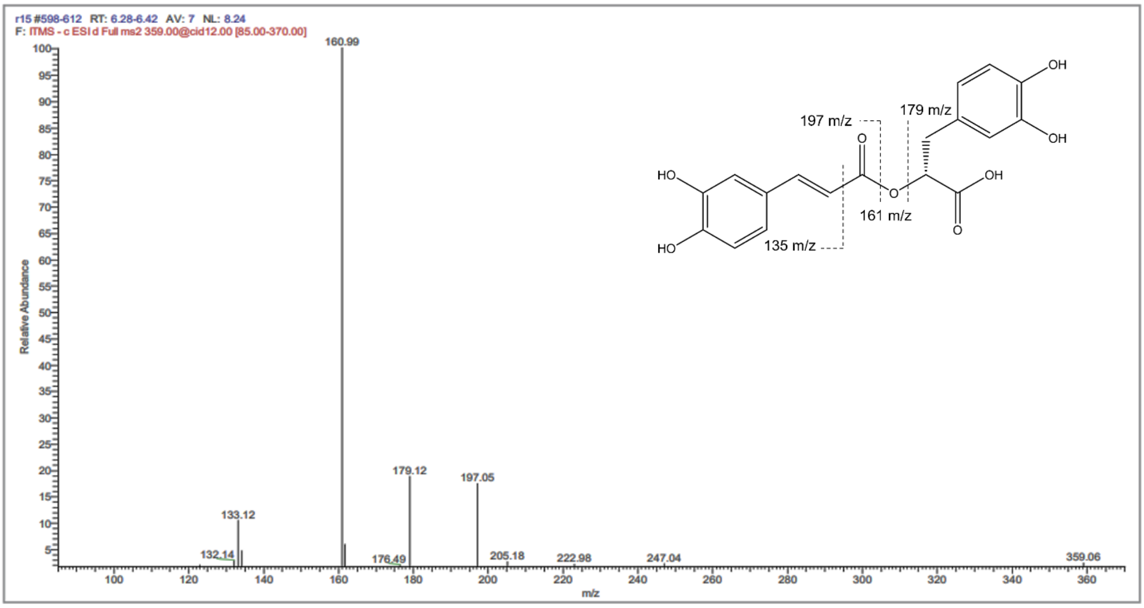

Figure S3. MS/MS spectrum of rosmarinic acid (**11**).

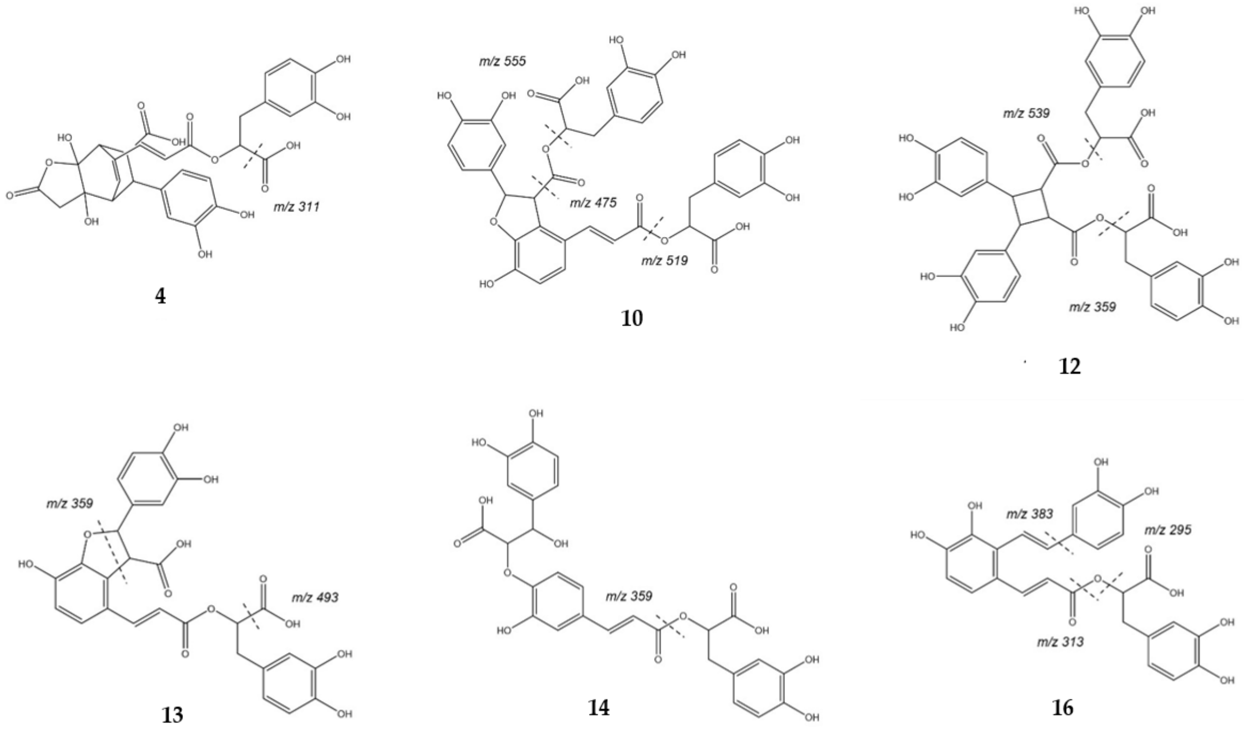

Figure S4. Fragmentation pathway of caffeic acid derivatives.

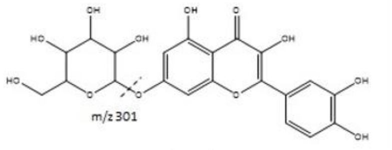

5

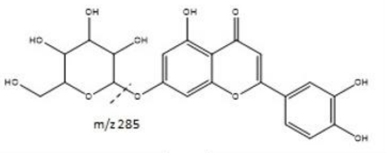

6

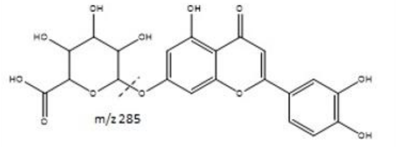

8

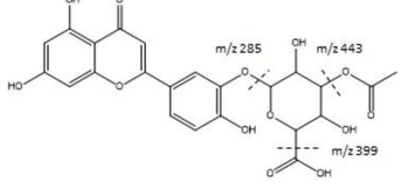

15

Figure S5. Fragmentation pathway of flavonoid glycosides.
